# Controllable dynamics of a dissipative two-level system

**DOI:** 10.1038/s41598-021-86553-z

**Published:** 2021-03-30

**Authors:** Wei Wu, Ze-Zhou Zhang

**Affiliations:** Lanzhou Center for Theoretical Physics, Key Laboratory of Theoretical Physics of Gansu Province, Lanzhou University, Lanzhou, 730000 Gansu China

**Keywords:** Quantum information, Theoretical physics, Quantum optics

## Abstract

We propose a strategy to modulate the decoherence dynamics of a two-level system, which interacts with a dissipative bosonic environment, by introducing an ancillary degree of freedom. It is revealed that the decay rate of the two-level system can be significantly suppressed under suitable steers of the assisted degree of freedom. Our result provides an alternative way to fight against decoherence and realize a controllable quantum dissipative dynamics.

## Introduction

A microscopic quantum system inevitably interacts with its surrounding environment, which generally results in decoherence^1–3^. Such decoherence process is responsible for the deterioration of quantumness and is commonly accompanied by energy or information dissipation. In this sense, how to prevent or avoid decoherence is of importance for any practical and actual quantum technology aimed at manipulating, communicating, or storing information. Furthermore, understanding decoherence in itself is one of the most fundamental issues in quantum mechanics, since it is closely associated with the quantum–classical transition^4^.

Up to now, various strategies have been proposed to suppress decoherence. For example, (1) the theory of decoherence-free subspace^5–7^, in which the quantum system undergoes a unitary evolution irrespective of environment’s influence; (2) dynamical decoupling pulse technique^8–10^, which aims at eliminating the unwanted system-environment coupling by a train of instantaneous pulses; (3) quantum Zeno effect^11–13^, which can inhibit the decay of a unstable quantum state by repetitive measurements; and (4) the bound-state-based mechanism scheme^14–17^, which can completely suppress decoherence and generate a dissipationless dynamics in the long-time regime. Each method has its own merit and corresponding weakness. For example, one needs to optimize the shapes and the intervals of pulses when using the dynamical decoupling pulse technique. Such optimization requires an elaborate operation as well as a great deal of experience. Here, we propose a simple scheme, which is more practical than the above methods. We believe that any alternative approach would be beneficial for us to achieve a reliable quantum processing in a noisy environment.

In this paper, we propose an efficient scheme to obtain a controllable dynamics of a two-level system (TLS), which interacts with a dissipative bosonic environment. An ancillary single-mode harmonic oscillator (HO), which acts as a steerable degree of freedom, is coupled to the TLS to modulate its decoherence dynamics^18–21^. We find the decay of the TLS can be suppressed via adjusting the parameters of the assisted HO. We also demonstrate the single-mode HO can be equivalently replaced by a periodic driving field or a multi-mode bosonic reservoir, which can likewise achieve the effect of decoherence-suppression. Moreover, we numerically confirm our steer scheme can be generalized to a more general quantum dissipative system, in which the TLS-environment coupling is strong and the so-called counter-rotating-wave terms are included.

## Results

### Controllable dissipative dynamics

Let us consider a TLS interacts with a dissipative bosonic environment. To achieve a tunable reduced dynamics of the TLS, we add an ancillary single-mode HO, which serves as a controllable degree of freedom to modulate the dynamical behaviour of the TLS. The whole system can be described as follows (throughout the paper, we set $\hbar =k_{\mathrm {B}}=c=1$)^18–21^

1 $$H = \frac{1}{2}\epsilon \sigma _{z} + \omega _{0} a^{\dag } a + \frac{1}{2}g_{0} \sigma _{z} (a^{\dag} + a) + \sum\limits_{k} {\omega _{k} } b_{k}^{\dag} b_{k} + \sum\limits_{k} {g_{k} } (\sigma _{ - } b_{k}^{\dag} + \sigma _{ + } b_{k} ),$$

where $\sigma _{\pm }\equiv \frac{1}{2}(\sigma _{x}\pm i\sigma _{y})$ with $\sigma _{x,y,z}$ being the standard Pauli operators, $\epsilon$ is the transition frequency of the TLS, $a^{\dagger }$ and *a* are creation and annihilation operators of the assisted HO with frequency $\omega _{0}$, and the parameter $g_{0}$ quantifies the coupling strength between the TLS and the HO. $b_{k}^{\dagger }$ and $b_{k}$ are creation and annihilation operators of the *k*th environmental mode with frequency $\omega _{k}$, respectively, and the TLS-environment coupling strengthes are denoted by $g_{k}$. The rotating wave approximation has already been made in the TLS-environment interaction term. Generally, it is very convenient to encode the frequency dependence of the interaction strengths in the spectral density $J(\omega )$, which is defined by $J(\omega )\equiv \sum _{k}g_{k}^{2}\delta (\omega -\omega _{k})$. By doing so, the characteristic of the environment is now completely determined by $J(\omega )$. In this work, the spectral density is characterized by the following Lorentzian form

2 $$J(\omega )=\frac{1}{\pi }\frac{\alpha \omega _{c}}{(\omega -\epsilon )^{2}+\omega _{c}^{2}},$$

where $\alpha$ is a coupling constant, and $\omega _{c}$ is a cutoff frequency. Instead of identifying the values of $g_{k}$ and $\omega _{k}$, we indicate the values of $\alpha$ and $\omega _{c}$ when dealing with the dissipative dynamics.

To obtain the dynamics of the dissipative TLS in an analytical form, we first apply a polaron transformation^22,23^ to the original Hamiltonian *H* as ${\tilde{H}}=e^{S}He^{-S}$, where the generator *S* is defined by $S=\frac{g_{0}}{2\omega _{0}}\sigma _{z}(a^{\dagger }-a)$. The transformed Hamiltonian can be expressed as

3 $${\tilde{H}}=\frac{1}{2}\epsilon \sigma _{z}+\omega _{0}a^{\dagger }a+\sum _{k}\omega _{k}b_{k}^{\dagger }b_{k}+\sum _{k}g_{k} {(}\sigma _{-}b_{k}^{\dagger }e^{-\zeta }+\mathrm {H}.\mathrm {c}. {)}-\frac{g_{0}^{2}}{4\omega _{0}},$$

where $\mathrm {H}.\mathrm {c}.$ denotes Hermitian conjugate and $\zeta \equiv \frac{g_{0}}{\omega _{0}}(a^{\dagger }-a)$. One can see the last term in the above expression is just a constant, which just induces a trivial dynamical phase and would not influence the reduced dynamical behaviour of the TLS. Thus, we will drop it from now on.

We employ the quantum master equation approach to investigate the reduced dynamics of the TLS. In the polaron representation, the second-order approximate quantum master equation reads^24^

4 $$\frac{d}{dt}{\tilde{\rho }}^{\mathrm {I}}_{\mathrm {s}}(t)=-\int _{0}^{t}d\tau {\mathrm {Tr}}_{\mathrm {ab}}\left\{ [{\tilde{H}}_{\mathrm {i}}(t),[{\tilde{H}}_{\mathrm {i}}(\tau ),{\tilde{\rho }}^{\mathrm {I}}_{\mathrm {tot}}(\tau )]]\right\} ,$$

where ${\tilde{\rho }}^{\mathrm {I}}_{\mathrm {s}}(t)\equiv e^{it{\tilde{H}}_{\mathrm {s}}}{\tilde{\rho }}_{\mathrm {s}}(t)e^{-it{\tilde{H}}_{\mathrm {s}}}$ with ${\tilde{H}}_{\mathrm {s}}\equiv \frac{1}{2}\epsilon \sigma _{z}$ is the reduced density operator in interaction picture, ${\tilde{H}}_{\mathrm {i}}(t)\equiv e^{it{\tilde{H}}_{0}}{\tilde{H}}_{\mathrm {i}}e^{-it{\tilde{H}}_{0}}$ with ${\tilde{H}}_{0}\equiv {\tilde{H}}_{\mathrm {s}}+{\tilde{H}}_{\mathrm {a}}+{\tilde{H}}_{\mathrm {b}}$, ${\tilde{H}}_{\mathrm {a}}\equiv \omega _{0} a^{\dagger }a$, ${\tilde{H}}_{\mathrm {b}}\equiv \sum _{k}\omega _{k}b_{k}^{\dagger }b_{k}$ and ${\tilde{H}}_{\mathrm {i}}\equiv \sum _{k}g_{k}(\sigma _{-}b_{k}^{\dagger }e^{-\zeta }+\mathrm {H}.\mathrm {c}.)$ is the interaction Hamiltonian in interaction picture. If both the TLS-HO and TLS-environment couplings are weak, one can safely adopt the Born approximation ${\tilde{\rho }}_{\mathrm {tot}}^{\mathrm {I}}(\tau )\simeq {\tilde{\rho }}^{\mathrm {I}}_{\mathrm {s}}(\tau )\otimes {\tilde{\rho }}_{\mathrm {a}}(0)\otimes {\tilde{\rho }}_{\mathrm {b}}(0)$. In this paper, we assume ${\tilde{\rho }}_{\mathrm {a}}(0)=|0_{\mathrm {a}}\rangle \langle 0_{\mathrm {a}}|$ and ${\tilde{\rho }}_{\mathrm {b}}(0)=\bigotimes _{k}|0_{\mathrm {b}}^{k}\rangle \langle 0_{\mathrm {b}}^{k}|$, where $|0_{\mathrm {a}}\rangle$ ($|0_{\mathrm {b}}^{k}\rangle$) is the Fock vacuum state of the single-mode HO (*k*-th bosonic environmental mode). The effect of non-Markovianity has been incorporated into the convolution terms. Such convolution terms mean the evolution of $\rho _{\mathrm {s}}(t)$ depends on $\rho _{\mathrm {s}}(\tau )$ at all the earlier times $0<\tau <t$, implying the memory effect from the environment has been considered. It should be emphasized that one can further use the Markov approximation by neglecting retardation in the integration of Eq. (4), namely ${\tilde{\rho }}^{\mathrm {I}}_{\mathrm {s}}(\tau )$ is replaced by ${\tilde{\rho }}^{\mathrm {I}}_{\mathrm {s}}(t)$. Our treatment is beyond such over-simplified Markovian approximation.

After some trivial algebra, we find the expression of ${\tilde{H}}_{\mathrm {i}}(t)$ is given by ${\tilde{H}}_{\mathrm {i}}(t)=\sum _{k}g_{k}[e^{-it(\epsilon -\omega _{k})}\sigma _{-}b_{k}^{\dagger }e^{-\zeta (t)}+\mathrm {H}.\mathrm {c}.]$, where $\zeta (t)\equiv e^{it\omega _{0}a^{\dagger }a}\zeta e^{-it\omega _{0}a^{\dagger }a}$. Substituting this expression of ${\tilde{H}}_{\mathrm {i}}(t)$ into the quantum master equation, namely Eq. (4), we have

5 $$\frac{d}{dt}{\tilde{\rho }}_{\mathrm {s}}^{\mathrm {I}}(t)=-\int _{0}^{t}d\tau \left\{ \sum _{k}g_{k}^{2}e^{i(\epsilon -\omega _{k})(t-\tau )}{\mathfrak {S}}(t-\tau ) {[}\sigma _{+}\sigma _{-}{\tilde{\rho }}^{\mathrm {I}}_{\mathrm {s}}(\tau )-\sigma _{-}{\tilde{\rho }}^{\mathrm {I}}_{\mathrm {s}}(\tau )\sigma _{+} {]}+\mathrm {H}.\mathrm {c}.\right\} ,$$

where ${\mathfrak {S}}(t-\tau )\equiv \langle 0_{\mathrm {a}}|e^{\zeta (t)}e^{-\zeta (\tau )}|0_{\mathrm {a}}\rangle$ is a dynamical modulation function. The exact expression of ${\mathfrak {S}}(t-\tau )$ can be derived by making use of the technique of Feynman disentangling of operators^21,25^. One can find

6 $${\mathfrak {S}}(t-\tau )=e^{-\lambda }\sum _{l=0}^{\infty }\frac{\lambda ^{l}}{l!}e^{-il\omega _{0}(t-\tau )},$$

where $\lambda \equiv (g_{0}/\omega _{0})^{2}$ is a steerable parameter completely determined by the ancillary HO. The dynamical modulation function ${\mathfrak {S}}(t-\tau )$ fully characterizes the influence of the single-mode HO on the reduced dynamics of the dissipative TLS.

### Non-equilibrium dynamics of population difference

Starting from Eq. (5), one can extract the equation of motion for the matrix components of the TLS, i.e., ${\tilde{\rho }}^{\mathrm {I}}_{\mathrm {jj'}}(t)\equiv \langle{ \mathrm {j}}|{\tilde{\rho }}^{\mathrm {I}}_{\mathrm {s}}(t)|\mathrm {j}'\rangle$ with $\mathrm {j},\mathrm {j}'=e,g$, where $|e\rangle$ and $|g\rangle$ are the eigenstates of $\sigma _{z}$. Meanwhile, due to the fact that ${\tilde{\rho }}^{\mathrm {I}}_{\mathrm {ee}}(t)={\tilde{\rho }}_{\mathrm {ee}}(t)$, we derived the following integro-differential equation for ${\tilde{\rho }}_{\mathrm {ee}}(t)$ in Schrödinger picture

7 $$\frac{d}{dt}{\tilde{\rho }}_{\mathrm {ee}}(t)=-\int _{0}^{t}d\tau e^{-\lambda }\sum _{l=0}^{\infty }\frac{\lambda ^{l}}{l!}\sum _{k}g_{k}^{2} {[}e^{i(\epsilon -\omega _{k}-l\omega _{0})(t-\tau )}{\tilde{\rho }}_{\mathrm {ee}}(\tau )+\mathrm {C}.\mathrm {c}. {]},$$

where $\mathrm {C}.\mathrm {c}.$ denotes complex conjugate. With the help of spectral density, one can replace the discrete summation in the above equation by a continuous integrand, i.e., $\sum _{k}g_{k}^{2}e^{-i\omega _{k}t}\rightarrow \int _{0}^{\infty }d\omega J(\omega )e^{-i\omega t}$. For the Lorentzian spectral density considered in this paper, the integrand can be greatly simplified by extending the integration range of $\omega$ from $[0,+\infty )$ to $(-\infty ,+\infty )$. Such approximation has been widely employed in several previous studies^1,15,26^ and is acceptable when the bound state effect can be neglected in the weak TLS-environment coupling regime^15^. Then, we have

8 $$\frac{d}{dt}{\tilde{\rho }}_{\mathrm {ee}}(t)=-\int _{0}^{t}d\tau \alpha e^{-\lambda }\sum _{l=0}^{\infty }\frac{\lambda ^{l}}{l!}e^{-\omega _{c}(t-\tau )} {[}e^{-il\omega _{0}(t-\tau )}\rho _{\mathrm {ee}}(\tau )+\mathrm {C}.\mathrm {c}. {]}.$$

We shall solve the integro-differential equation in Eq. (8) by making use of Laplace transformation, which is defined by $f(z)={{\mathscr {L}}}[f(t)]\equiv \int _{0}^{\infty }dte^{-zt}f(t)$. After the Laplace transformation, we find ${\tilde{\rho }}_{\mathrm {ee}}(z)/{\tilde{\rho }}_{\mathrm {ee}}(0)=[z+\mu (z)]^{-1}$, where the Laplace-transformed kernel $\mu (z)$ is given by

9 $$\mu (z)={{\mathscr {L}}} \bigg{[}2\alpha e^{-\lambda }\sum _{l=0}^{\infty }\frac{\lambda ^{l}}{l!}\cos (l\omega _{0}t)e^{-\omega _{c}t} \bigg{]}=2\alpha e^{-\lambda }\sum _{l=0}^{\infty }\frac{\lambda ^{l}}{l!}\frac{z+\omega _{c}}{(z+\omega _{c})^{2}+l^{2}\omega _{0}^{2}}.$$

Thus, the expression of population difference in the polaron representation can be obtained via ${\tilde{P}}(t)\equiv {\mathrm {Tr}}_{\mathrm {s}}[\sigma _{z}{\tilde{\rho }}_{\mathrm {s}}(t)]=2{\tilde{\rho }}_{\mathrm {ee}}(t)-1$. Next, we need to transform ${\tilde{P}}(t)$ back to the original representation. Thanks to the fact $[\sigma _{z},S]=0$, the expression of population difference does not change by the polaron transformation, i.e., $P(t)={\tilde{P}}(t)$. Finally, we arrive at

10 $$P(t)=2{{\mathscr {L}}}^{-1} \left[\frac{{\tilde{\rho }}_{\mathrm {ee}}(0)}{z+\mu (z)} \right]-1,$$

where ${{\mathscr {L}}}^{-1}$ denotes inverse Laplace transformation, i.e. ${{\mathscr {L}}}^{-1}[f(z)]\equiv \frac{1}{2\pi i}\int _{\varsigma -i\infty }^{\varsigma +i\infty }dte^{zt}f(z)$. As long as the initial state is given, the dynamics of *P*(*t*) can be fully determined by Eq. (10). In this paper, the inverse Laplace transformation is numerically performed by making use of the Zakian method^27^, which uses a series of weight functions to approximate an arbitrary function’s inverse Laplace transform in time domain. It should be stressed that Eq. (10) only works in the regime where both $\alpha$ and $\lambda$ are small, namely $\alpha /\omega _{c}\ll 1$ and $\lambda \ll 1$, due to the Born and the second-order master equation approximations.

**Figure 1:**
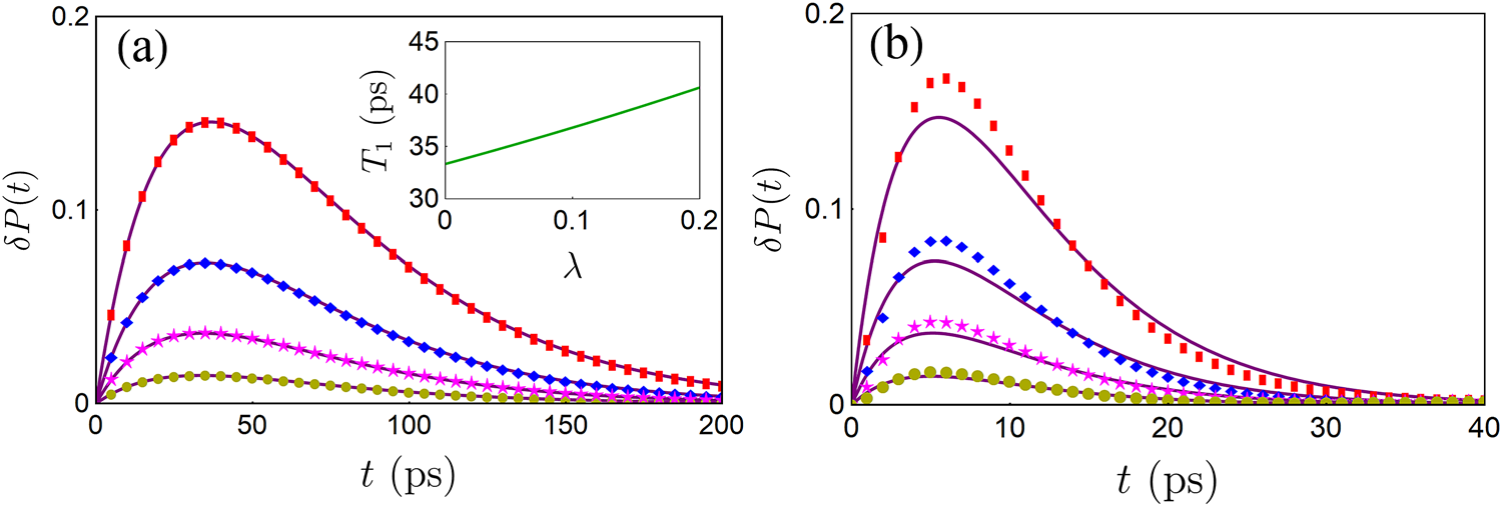
(**a**) $\delta P(t)$ is plotted as the function of time with different steer parameters: $\lambda =0.02$ (yellow circles), $\lambda =0.05$ (magenta stars), $\lambda =0.1$ (blue diamonds) and $\lambda =0.2$ (red squares). The purple solid lines are obtained from the Wigner–Weisskopf approximate expression of ${{\mathscr {P}}}(t)-P_{0}(t)$. The insert curve shows the relation between $T_{1}$ and $\lambda$. The initial state of the TLS is $|e\rangle \langle e|$, other parameters are chosen as $\omega _{0}=100\,{ \mathrm {cm}}^{-1}$, $\omega _{c}=10\, \mathrm {cm}^{-1}$ and $\alpha =0.15\, \mathrm {cm}^{-1}$. (**b**) The same with (**a**), but $\omega _{c}=1.5 \,\mathrm {cm}^{-1}$.

On the other hand, the sum of *l* in the expressions of $\mu (z)$ in Eq. (9) can be exactly worked out

11 $$\mu (z)=\frac{2\alpha e^{-\lambda }}{z+\omega _{c}}{\mathbf {F}} \left[\left\{ -\frac{iz}{\omega _{0}}-\frac{i\omega _{c}}{\omega _{0}},\frac{iz}{\omega _{0}}+\frac{i\omega _{c}}{\omega _{0}}\right\} ,\left\{ 1-\frac{\sqrt{-(z+\omega _{c})^{2}}}{\omega _{0}},1+\frac{\sqrt{-(z+\omega _{c})^{2}}}{\omega _{0}}\right\} ,\lambda \right],$$

where ${\mathbf {F}}[\{\mathrm {x}_{1},\mathrm {x}_{2},\ldots ,\mathrm {x}_{\mathrm {m}}\},\{\mathrm {y}_{1},\mathrm {y}_{2},\ldots ,\mathrm {y}_{\mathrm {n}}\},\mathrm {z}]$ is the generalized hypergeometric function^28^. If the TLS and the single-mode HO is completely decoupled, using Eq. (11), one can easily demonstrate $\lim _{\lambda \rightarrow 0}\mu (z)=2\alpha /(z+\omega _{c})$. In this special case, the inverse Laplace transformation in Eq. (10) can be analytically done and the expression of *P*(*t*) is then given by

12 $$P_{0}(t)\equiv \lim _{\lambda \rightarrow 0}P(t)=2e^{-\frac{1}{2}\omega _{c}t} \left[\cosh \left(\frac{1}{2}\Theta t \right)+\frac{\omega _{c}}{\Theta }\sinh \left(\frac{1}{2}\Theta t \right) \right]-1,$$

where $\Theta =\sqrt{\omega _{c}^{2}-8\alpha }$. This result reproduces the Eq. (10.51) in Ref.^1^.

### Decoherence time

In an approximate treatment, the density matrix components of the TLS commonly exhibit exponential decays, which are governed by the relaxation time $T_{1}$ and the dephasing time $T_{2}$ describing the evolution of $\rho _{\mathrm {ee}}(t)$ and $\rho _{\mathrm {eg}}(t)$, respectively. Thus, the decoherence time $T_{1,2}$ roughly reflects the characteristic of dissipative dynamics^29^. Here, we would like to evaluate the expression of the relaxation time $T_{1}$ and explore the influence of the assisted HO on the decoherence time.

Starting from Eq. (7), one can find

13 $${\tilde{\rho }}_{\mathrm {ee}}(t)=\frac{{\tilde{\rho }}_{\mathrm {ee}}(0)}{2\pi i}\int _{\varsigma -i\infty }^{\varsigma +i\infty } dz\frac{e^{zt}}{z+\mu _{+}(z)+\mu _{-}(z)},$$

where

14 $$\mu _{\pm }(z)=e^{-\lambda }\sum _{l=0}^{\infty }\frac{\lambda ^{l}}{l!}\sum _{k}\frac{g_{k}^{2}}{z\pm i(\epsilon -\omega _{k}-l\omega _{0})}.$$

Strictly speaking, the integration in Eq. (13) should be performed with the Bromwich path. However, in an approximate treatment, the Bromwich path can be changed to that on the real axis $-\infty<\varpi <\infty$ by a transform $z=i\varpi +0^{+}$^25,30–32^, where $0^{+}$ denotes a positive infinitesimal. Under such treatment, we find

15 $${\tilde{\rho }}_{\mathrm {ee}}(t)=\frac{{\tilde{\rho }}_{\mathrm {ee}}(0)}{2\pi i}\int _{-\infty }^{+\infty }d\varpi \frac{e^{i\varpi t}}{\varpi -i\mu _{+}(i\varpi +0^{+})-i\mu _{-}(i\varpi +0^{+})}.$$

Using the Sokhotski–Plemelj theorem

16 $$\frac{1}{x\pm i0^{+}}={\mathbb {P}}\frac{1}{x}\mp i\pi \delta (x),$$

we have $i\mu _{\pm }(i\varpi +0^{+})=\Sigma _{\pm }(\varpi )-i\Gamma _{\pm }(\varpi )$, where

17 $$\begin{aligned} \Sigma _{\pm }(\varpi )&=e^{-\lambda }\sum _{l=0}^{\infty }\frac{\lambda ^{l}}{l !} \sum _{k}\frac{g_{k}^{2}}{\varpi \pm (\epsilon -\omega _{k}-l\omega _{0})},~~\Gamma _{\pm }(\varpi )\nonumber \\&=\pi e^{-\lambda }\sum _{l=0}^{\infty }\frac{\lambda ^{l}}{l !}\sum _{k}g_{k}^{2}\delta [\varpi \pm (\epsilon -\omega _{k}-l\omega _{0})]. \end{aligned}$$

Thus, we finally arrive at

18 $${\tilde{\rho }}_{\mathrm {ee}}(t)=\frac{{\tilde{\rho }}_{\mathrm {ee}}(0)}{2\pi i}\int _{-\infty }^{+\infty }d\varpi \frac{e^{i\varpi t}}{[\varpi -\Sigma _{+}(\varpi )-\Sigma _{-}(\varpi )]+i[\Gamma _{+}(\varpi )+\Gamma _{-}(\varpi )]},$$

The pole of the above integrand can be approximately viewed as $\varpi _{0}+i\Gamma _{+}(\varpi _{0})+i\Gamma _{-}(\varpi _{0})$, where $\varpi _{0}$ is determined by $\varpi _{0}-\Sigma _{+}(\varpi _{0})-\Sigma _{-}(\varpi _{0})=0$. Then, the integration can be worked out by using the residue theorem and the result is ${\tilde{\rho }}_{\mathrm {ee}}(t)\simeq {\tilde{\rho }}_{\mathrm {ee}}(0)e^{i\varpi _{0}t}e^{-[\Gamma _{+}(\varpi _{0})+\Gamma _{-}(\varpi _{0})]t}$. In the weak-coupling regime, one can neglect the level shift induced by $\Sigma _{\pm }(\varpi )$^30–32^, which results in $\varpi _{0}\simeq 0$. Finally, the expression of $T_{1}$ can be further simplified to

19 $$\begin{aligned} T_{1}^{-1}\simeq&\sum _{\mathrm {k}=\pm }\Gamma _{\mathrm {k}}(0)=2\pi e^{-\lambda }\sum _{l=0}^{\infty }\frac{\lambda ^{l}}{l !} J(\epsilon -l\omega _{0})\nonumber \\ =&-\frac{i\alpha }{\omega _{0}}e^{-\lambda }(-\lambda )^{-\frac{i\omega _{c}}{\omega _{0}}} \left[(-\lambda )^{\frac{2i\omega _{c}}{\omega _{0}}}{\mathbf {G}} \left(-\frac{i\omega _{c}}{\omega _{0}},0,-\lambda \right)-{\mathbf {G}} \left(\frac{i\omega _{c}}{\omega _{0}},0,-\lambda \right) \right], \end{aligned}$$

where ${\mathbf {G}}(\mathrm {x},\mathrm {y}_{1},\mathrm {y}_{2})$ is the generalized incomplete gamma function^28^. Accordingly, the approximate expression of population difference is ${{\mathscr {P}}}(t)\simeq 2\exp (-t/T_{1})-1$. One can see $\lim _{\lambda \rightarrow 0}T_{1}^{-1}=2\pi J(\epsilon )$, which reproduces the well-known Wigner-Weisskopf decay rate without invoking the assisted HO^24^.

In Fig. 1, we plot the dynamics of $\delta P(t)\equiv P(t)-P_{0}(t)$, which can be regarded as a witness to the effectiveness of our scheme. If $\delta P(t)>0$, i.e., $P(t)>P_{0}(t)$, one can conclude that the decay of the population difference is slowed down when turning on the coupling between the TLS and the assisted HO. From Fig. 1, one can see $\delta P(t)$ can be increased by enhancing $\lambda$, which means the coherent dynamics of *P*(*t*) becomes more and more robust as $\lambda$ becomes larger. In this sense, by adjusting the parameters of the ancillary degree of freedom, we can achieve a controllable quantum dissipative dynamics. As comparisons, we also display ${{\mathscr {P}}}(t)-P_{0}(t)$. One can see from Fig. 1a that the results from the two different methods are in good agreement for the Markovian regime $\alpha /\omega _{c}\rightarrow 0$. However, in non-Markovian regime (see Fig. 1b), a deviation is found. We believe such deviation is induced by the non-Markovianity incorporated in our approach. These results demonstrate our steer scheme works well in both Markovian and non-Markovian cases. Moreover, in Fig. 1a, one can observe that the relaxation time can be effectively prolonged by increasing the value of $\lambda$. This result is consistent with our previous numerical simulations. Using the same method, we also find $T_{2}^{-1}=\frac{1}{2}T_{1}^{-1}$, which means the dephasing time can be lengthened by adjusting the parameter $\lambda$ as well. From the analytical expression of the decoherence time, we once again demonstrate the validity of our steer scheme.

### Generalizations

Next, we would like to show that the single-mode HO can be equivalently replaced by a periodic driving field or a multi-mode bosonic reservoir. Though the physical properties of these assisted degrees of freedom are completely different, the effect of decoherence-suppression remains unchanged. Moreover, we extend the single-mode-HO-based steer scheme to a more general quantum dissipative system with hierarchical equations of motion (HEOM) approach, in which the counter-rotating-wave terms are included.

#### Periodic driving field case

The assisted degree of freedom can be replaced by a periodic driving along the *z* direction. We can construct the following time-dependent Hamiltonian in which the TLS is engineered by a cosine driving term,

20 $$H(t)=\frac{1}{2}\epsilon \sigma _{z}+\frac{1}{2}A\cos (\Omega t)\sigma _{z}+\sum _{k}\omega _{k}b_{k}^{\dagger }b_{k}+\sum _{k}g_{k}{(}\sigma _{-}b_{k}^{\dagger }+\sigma _{+}b_{k}{)},$$

where *A* is the driving amplitude and $\Omega$ is the driving frequency. The dynamics of the whole system is governed by the Schrödinger equation $\partial _{t}|\psi (t)\rangle =-iH(t)|\psi (t)\rangle$. To handle the time-dependent term in the above Schrödinger equation, we apply a time-dependent transformation to $|\psi (t)\rangle$ as $|{\tilde{\psi }}(t)\rangle =e^{S_{t}}|\psi (t)\rangle$, where the time-dependent generator is given by $S_{t}=i\frac{A}{2\Omega }\sin (\Omega t)\sigma _{z}$^33,34^. Then, in the transformed representation, the dynamics of $|{\tilde{\psi }}(t)\rangle$ is governed by $\partial _{t}|{\tilde{\psi }}(t)\rangle =-i{\tilde{H}}(t)|{\tilde{\psi }}(t)\rangle$, where

21 $${\tilde{H}}(t)=e^{S_{t}}[H(t)-i\partial _{t}]e^{-S_{t}}=\frac{1}{2}\epsilon \sigma _{z}+\sum _{k}\omega _{k}b_{k}^{\dagger }b_{k}+\sum _{k}g_{k}{[}\sigma _{-}e^{-i\phi (t)}b_{k}^{\dagger }+\mathrm {H}.\mathrm {c}. {]},$$

with $\phi (t)=\frac{A}{\Omega }\sin (\Omega t)$. If the driving frequency is sufficiently high, the time-dependent Hamiltonian ${\tilde{H}}(t)$ can be approximately replaced a much simpler, undriven effective Hamiltonian^33,34^. To be more specific, using the Jacobi–Anger identity

22 $$e^{ix\sin \beta }=\sum _{n=-\infty }^{\infty }{{\mathscr {J}}}_{n}(x)e^{in\beta },$$

where ${{\mathscr {J}}}_{n}(x)$ are Bessel functions of the first kind^28^, one can only retain the lowest order term and neglect all the other higher-order terms in $e^{\pm i\phi (t)}$, namely,

23 $$\exp \bigg{[}\pm i\frac{A}{\Omega }\sin (\Omega t) \bigg{]}\simeq {{\mathscr {J}}}_{0} \bigg{(}\frac{A}{\Omega } \bigg{)}.$$

Then, one can obtain an effective interaction Hamiltonian $H_{\mathrm {i}}^{\mathrm {eff}}(t)=\sum _{k}{\check{g}}_{k}(\sigma _{-}e^{-i\epsilon t}b_{k}^{\dagger }e^{i\omega _{k}t}+\mathrm {H}.\mathrm {c}.)$, where the renormalized coupling strength is defined by ${\check{g}}_{k}={{\mathscr {J}}}_{0}(A/\Omega )g_{k}$. Compared with that of the undriven case, one can see the periodic driving field actually renormalizes the coupling constant $\alpha$ in the spectral density, i.e., $\alpha \rightarrow {\check{\alpha }}={{\mathscr {J}}}_{0}(A/\Omega )^{2}\alpha$. Considering the fact that $0\le {{\mathscr {J}}}_{0}(A/\Omega )^{2}\le 1$, then ${\check{\alpha }}\le \alpha$. This result is quite similar to the HO assisted case in which the coupling strengthes are renormalized as $g_{k}^{2}\rightarrow g_{k}^{2}{\mathfrak {S}}(t-\tau )$ (see Eq. 5). Thus, the periodic driving field is able to facilitate a robust coherent dynamics as well. More importantly, due to the fact that the periodic driving technique has been widely used in the experiments of cold atom systems, it is more friendly from experimental perspective. In fact, a similar periodic driving field has been used to control the dynamics of quantum circuits in the recent experiment^34^.

**Figure 2:**
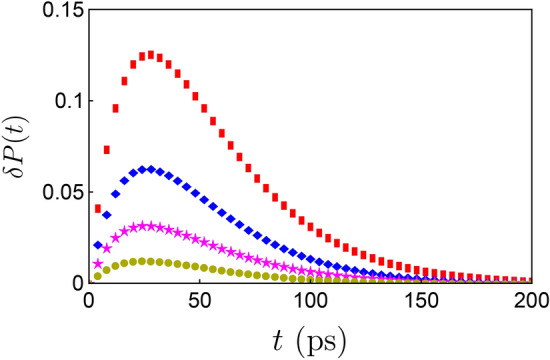
$\delta P(t)$ is plotted as the function of time with different steer parameters: $\Lambda =0.02$ (yellow circles), $\Lambda =0.05$ (magenta stars), $\Lambda =0.1$ (blue diamonds) and $\Lambda =0.2$ (red squares). The initial state of the TLS is $|e\rangle \langle e|$, other parameters are chosen as $\eta =30\, \mathrm {cm}^{-1}$, $\omega _{c}=5\, \mathrm {cm}^{-1}$ and $\alpha =0.1\, \mathrm {cm}^{-1}$.

#### Multi-mode bosonic reservoir case

Our scheme can be also generalized to the case where the assisted degree of freedom is a multi-mode bosonic reservoir. The whole Hamiltonian of the modulated system in this situation is given by

24 $$H=\frac{1}{2}\epsilon \sigma _{z}+\sum _{j}\nu _{j}a_{j}^{\dagger }a_{j}+\frac{1}{2}\sum _{j}\kappa _{j}\sigma _{z}{(}a_{j}^{\dagger }+a_{j} {)}+\sum _{k}\omega _{k}b_{k}^{\dagger }b_{k}+\sum _{k}g_{k} {(}\sigma _{-}b_{k}^{\dagger }+\sigma _{+}b_{k} {)},$$

where $a_{j}^{\dagger }$ and $a_{j}$ are creation and annihilation operators of the *j*th assisted bosonic mode with frequency $\nu _{j}$, respectively, the coupling strengths between the TLS and assisted reservoir are characterized by $\kappa _{j}$. The spectral density of the assisted reservoir is then defined by $\varrho (\nu )\equiv \sum _{j}\kappa _{j}^{2}\delta (\nu -\nu _{j})$. Similar to the single-mode HO case, we apply a polaron transformation to Eq. (24) as ${\tilde{H}}=e^{G}He^{-G}$, where the generator *G* is given by

25 $$G=\sum _{j}\frac{\kappa _{j}}{2\nu _{j}}\sigma _{z} {(}a_{j}^{\dagger }-a_{j} {)}.$$

Then, the transformed Hamiltonian ${\tilde{H}}$ is given by

26 $${\tilde{H}}=\frac{1}{2}\epsilon \sigma _{z}+\sum _{j}\nu _{j}a_{j}^{\dagger }a_{j}+\sum _{k}\omega _{k}b_{k}^{\dagger }b_{k}+\sum _{k}g_{k} {(}\sigma _{-}b_{k}^{\dagger }e^{-\xi }+\sigma _{+}b_{k}e^{\xi } {)},$$

where $\xi =\sum _{j}\frac{\kappa _{j}}{\nu _{j}}(a_{j}^{\dagger }-a_{j})$. Assuming ${\tilde{\rho }}_{\mathrm {ab}}(0)={\tilde{\rho }}_{\mathrm {a}}(0)\otimes {\tilde{\rho }}_{\mathrm {b}}(0)$ with ${\tilde{\rho }}_{\mathrm {a}}(0)=\bigotimes _{j}|0_{\mathrm {a}}^{j}\rangle \langle 0_{\mathrm {a}}^{j}|$, ${\tilde{\rho }}_{\mathrm {b}}(0)=\bigotimes _{k}|0_{\mathrm {b}}^{k}\rangle \langle 0_{\mathrm {b}}^{k}|$ and using the same quantum master equation approach displayed in single-mode HO case, one can find

27 $$\frac{d}{dt}{\tilde{\rho }}_{\mathrm {ee}}(t)=-\int _{0}^{t}d\tau \sum _{k}g_{k}^{2} {[}e^{i(\epsilon -\omega _{k})(t-\tau )}{\mathfrak {G}}(t-\tau ){\tilde{\rho }}_{\mathrm {ee}}(\tau )+\mathrm {H}.\mathrm {c}. {]},$$

where the dynamical modulation function is given by

28 $${\mathfrak {G}}(t)=\prod _{j}\exp \left(-\frac{\kappa _{j}^{2}}{\nu _{j}^{2}} \right)\sum _{l=0}^{\infty }\frac{1}{l!} \left(\frac{\kappa _{j}^{2}}{\nu _{j}^{2}} \right)^{l}e^{-il\nu _{j}t}=\exp \left[\sum _{j}\frac{\kappa _{j}^{2}}{\nu _{j}^{2}} \left(e^{-i\nu _{j}t}-1 \right) \right].$$

Assuming $\varrho (\nu )$ has a super-Ohmic spectral density with a Lorentz-type cutoff form, i.e.,

29 $$\varrho (\nu )=\frac{1}{\pi }\frac{\chi \nu ^{2}}{\nu ^{2}+\eta ^{2}},$$

where $\chi$ is the coupling constant and $\eta$ is the cutoff frequency. Then, ${\mathfrak {G}}(t)$ has a very simple expression

30 $${\mathfrak {G}}(t)\simeq \exp \bigg{[}\int _{-\infty }^{\infty }d\varepsilon \frac{\varrho (\nu )}{\nu ^{2}} {(}e^{-i\nu t}-1 {)} \bigg{]}=\exp {(}\Lambda e^{-\eta t}-\Lambda {)}=e^{-\Lambda }\sum _{l=0}^{\infty }\frac{\Lambda ^{l}}{l!}e^{-l\eta t},$$

where $\Lambda =\chi /\eta$. Compared with that of Eq. (6), one can see $\Lambda$ plays the same role with that of $\lambda$. Following the same process exhibited in single-mode case, one can find the expression of population difference *P*(*t*) is almost the same with Eq. (10), the only difference is the expression of $\mu (z)$ should be replaced by

31 $$\mu (z)=2\alpha e^{-\Lambda }\sum _{l=0}^{\infty }\frac{\Lambda ^{l}}{l!}\frac{1}{z+\omega _{\mathrm {c}}+l\eta }.$$

In Fig. 2, we display the dynamics of $\delta P(t)$ in the case where the assisted degree of freedom is a multi-mode bosonic reservoir. One can see the decay of *P*(*t*) can be inhibited due to the interplay between the TLS and the additional degrees of freedom. Similar to single-mode HO case, the decay rate can be further reduced by increasing the value of $\Lambda$. Our result is in agreement with that of Ref.^35^ in which authors use a stochastic dephasing fluctuation to suppress the relaxation processes of two-level and three-level atomic systems. The physical picture behind this phenomenon is the ancillary degree of freedom effectively modifies the property of original environment acting on the TLS, which gives rise to this decoherence-suppression effect. Similar results have been also reported in several previous studies^21,36–38^.

**Figure 3:**
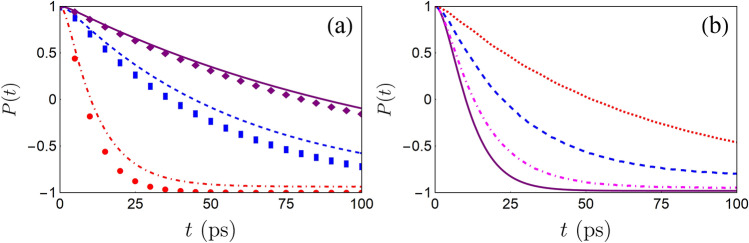
(**a**) *P*(*t*) with different coupling constants: $\alpha =0.0025~\mathrm {cm}^{-1}$ (purple solid line is the numerical result from HEOM method, purple diamonds are analytical results from Eq. (10)), $\alpha =0.005~\mathrm {cm}^{-1}$ (blue dashed line is the numerical result from HEOM method, blue squares are analytical results from Eq. (10)) and $\alpha =0.025~\mathrm {cm}^{-1}$ (red dotdashed line is the numerical result from HEOM method, red circles are analytical results from Eq. (10)). Other parameters are chosen as $\epsilon =1.5~\mathrm {cm}^{-1}$, $\lambda =0.05$, $\omega _{0}=5~\mathrm {cm}^{-1}$ and $\omega _{c}=0.5~\mathrm {cm}^{-1}$. (**b**) The dynamics *P*(*t*) from the HEOM method with different tunable parameters: $\lambda =0$ (purple solid line), $\lambda =0.15$ (magenta dotdashed line), $\lambda =0.25$ (blue dashed line) and $\lambda =1$ (red dotted line). Other parameters are chosen as $\alpha =0.025~\mathrm {cm}^{-1}$, $\epsilon =1.5~\mathrm {cm}^{-1}$, $\omega _{0}=2.5~\mathrm {cm}^{-1}$ and $\omega _{c}=0.35~\mathrm {cm}^{-1}$.

#### HEOM treatment

We have demonstrated that the decoherence of the TLS can be effectively suppressed by introducing an auxiliary single-mode HO. However, this conclusion is obtained under the weak-coupling and rotating-wave approximations. Going beyond these limitations, we next consider a more general quantum dissipative system

32 $$H=\frac{1}{2}\epsilon \sigma _{z}+\omega _{0}a^{\dagger }a+g_{0}\sigma _{z}(a^{\dagger }+a)+\sum _{k}\omega _{k}b_{k}^{\dagger }b_{k}+\sum _{k}g_{k}\sigma _{x} {(}b_{k}^{\dagger }+b_{k} {)}.$$

Compared with Eq. (1), the counter-rotating-wave terms have been incorporated in the above Hamiltonian.

To handle the reduced dynamics without the rotating-wave approximation, we employ a purely numerical method, the HEOM approach^39–43^, to obtain the exact reduced dynamics of the TLS. The HEOM can be viewed as a bridge connecting the standard Schrödinger equation, which is exact but commonly hard to solve directly, and a set of ordinary differential equations, which can be treated numerically by using the well-developed Runge–Kutta algorithm. Without invoking the Born, weak-coupling and rotating-wave approximations, the HEOM can provide a rigorous numerical result as long as the initial state of the whole system is a system-environment separable state. To realize the traditional HEOM algorithm, it is necessary that the zero-temperature environmental correlation function $C(t)=\int d\omega J(\omega )e^{-i\omega t}$ can be (or at least approximately) written as a finite sum of exponentials^43,44^. Fortunately, one can easily demonstrate that $C(t)=\alpha e^{-(\omega _{c}+i\epsilon )t}$ for the Lorentzian spectral density considered in this paper. Then, following the procedure shown in Refs.^43,44^, one can obtain the following hierarchy equations

33 $$\frac{d}{dt}\rho _{{\vec {\ell }}}(t)= \Big{(}-iH_{\mathrm {sa}}^{\times }-{\vec {\ell }}\cdot {\vec {\upsilon }} \Big{)}\rho _{{\vec {\ell }}}(t)+\Phi \sum _{p=1}^{2}\rho _{{\vec {\ell }}+{\vec {e}}_{p}}(t)+\sum _{p=1}^{2}\ell _{p}\Psi _{p}\rho _{{\vec {\ell }}-{\vec {e}}_{p}}(t),$$

where $\rho _{{\vec {\ell }}={\vec {0}}}(t)$ is the reduced density operator of the TLS plus the HO, $\rho _{{\vec {\ell }}\ne {\vec {0}}}(t)$ are auxiliary operators introduced in HEOM algorithm,

34 $$H_{\mathrm {sa}}=\frac{1}{2}\epsilon \sigma _{z}+\omega _{0}a^{\dagger }a+g_{0}\sigma _{z}(a^{\dagger }+a),$$

${\vec {\ell }}=(\ell _{1},\ell _{2})$ is a two-dimensional index, ${\vec {e}}_{1}=(1,0)$, ${\vec {e}}_{2}=(0,1)$, and ${\vec {\upsilon }}=(\omega _{c}-i\epsilon ,\omega _{c}+i\epsilon )$ are two-dimensional vectors, two superoperators $\Phi$ and $\Psi _{p}$ are defined by

35 $$\Phi X=-i[\pmb {\sigma }_{x},X],~~~\Psi _{p}X=\frac{i}{2}\alpha (-1)^{p}\{\pmb {\sigma }_{x},X\}-\frac{i}{2}\alpha [\pmb {\sigma }_{x},X],$$

where $\pmb {\sigma }_{x}=\sigma _{x}\otimes {\mathbf {1}}_{\mathrm {a}}$ with ${\mathbf {1}}_{\mathrm {a}}$ being an identity operator of the HO, $[X,Y]\equiv XY-YX$ and $\{X,Y\}\equiv XY+YX$.

The initial-state conditions of the auxiliary operators are given by $\rho _{{\vec {\ell }}={\vec {0}}}(0)=\rho _{\mathrm {sa}}(0)$ and $\rho _{{\vec {\ell }}\ne {\vec {0}}}(0)=0$, where ${\vec {0}}=(0,0)$ is a two-dimensional zero vector. For numerical simulations, we need to truncate the number of hierarchical equations for a sufficiently large integer $\ell _{c}$, which can guarantee the numerical convergence. All the terms of $\rho _{{\vec {\ell }}}(t)$ with $\ell _{1}+\ell _{2}>\ell _{c}$ are set to be zero, and the terms of $\rho _{{\vec {\ell }}}(t)$ with $\ell _{1}+\ell _{2}\le \ell _{c}$ form a closed set of differential equations. Technically speaking, the single-mode HO is a $\infty$-dimensional matrix in its Fock state basis $\{|0_{\mathrm {a}}\rangle ,|1_{\mathrm {a}}\rangle ,|2_{\mathrm {a}}\rangle ,...\}$. Thus, the size of HO should be truncated in practical simulations. In this paper, we approximately regard the HO as a $10\times 10$ matrix due to the limitation of our computation resource, and we have checked that the reduced dynamics of the TLS remains unchanged by further increasing the size of the assisted degree of freedom.

Assuming $\rho _{\mathrm {sa}}(0)=|e\rangle \langle e|\otimes e^{-S}|0_{\mathrm {a}}\rangle \langle 0_{\mathrm {a}}|e^{S}$, the reduced density operator of the TLS is obtained by partially tracing out of the degree of freedom of the HO from $\rho _{{\vec {\ell }}={\vec {0}}}(t)$, i.e. $\rho _{\mathrm {s}}(t)=\mathrm {Tr}_{\mathrm {a}}[\rho _{{\vec {\ell }}={\vec {0}}}(t)]$. Figure 3 shows our numerical results obtained by the HEOM approach. It is found that the result from Eq. (10) is in qualitative agreement with those of the numerical HEOM method in weak-coupling regime. However, when coupling becomes strong, the counter-rotating-wave terms lead to a deviation. This result is physically understandable, because the the counter-rotating-wave terms are neglectable in weak-coupling case. Moreover, one can clearly see the decay of *P*(*t*) is suppressed by switching on the TLS-HO coupling. As $\lambda$ increases, the effect of coherence-preservation becomes more noticeable. This result indicates that our steer scheme can be generalized to the non-rotating-wave approximation case, which greatly extends the scope of validity of our steer scheme.

## Discussion

In our theoretical scheme, the inclusion of the single-mode HO can considerably protect the quantum coherence, and the value of $\lambda$ plays a crucial role in our recipe. How to obtain a relatively large value of $\lambda$ is the main difficulty in realizing our control scheme from an experimental perspective. Fortunately, the research of light-matter interaction has made a great progress in experiment. Nowadays, researchers are able to simulate the quantum Rabi model, whose Hamiltonian is described by $H_{\mathrm {Rabi}}=-\frac{1}{2}(\Delta \sigma _{x}+\epsilon \sigma _{z})+\omega _{\mathrm {o}}(a^{\dagger }a+\frac{1}{2})+g\sigma _{z}(a^{\dagger }+a)$, in the ultra-strong-coupling and the deep-strong-coupling regimes. For example, by making use of a superconducting flux qubit and an LC oscillator via Josephson junctions, Yoshihara et al. have experimentally realized a superconducting circuits with the value of $\lambda$ ranging from 0.72 to 1.34 and $g/\Delta \gg 1$^45^. These experimental progresses can provide a strong support to our steer scheme in realistic physical systems.

In conclusion, we propose a strategy to realize a controllable dynamics of a dissipative TLS with the help of an assisted degrees of freedom, which can be a single-mode HO, a periodic driving field or a multi-mode bosonic reservoir. Via adjusting the parameters of the assisted degree of freedom, we find the decoherence rate of the TLS can be significantly suppressed regardless of whether the counter-rotating-wave terms are taken into account. The physical picture behind this phenomenon is because the decays induced by parallel interaction (caused by the assisted degrees of freedom) and perpendicular interaction (intrinsically appeared in the original Hamiltonian) compete with each other, which effectively modifies the decoherence induced by the perpendicular interaction and gives rise to this coherence-preserve effect. Though our results are achieved in a Lorentzian environment at zero temperature, it would be very interesting to generalize our steer scheme to some more general situations by using the HEOM method, which has been extended to explore the dissipative dynamics in finite-temperature environment described by an arbitrary spectral density function^43,44,46–48^. Finally, due to the generality of the dissipative TLS model, we expect our result to be of interest for some applications in quantum optics and quantum information.

## References

[1] Breuer HP, Petruccione F (2002). The Theory of Open Quantum Systems.

[2] Weiss U (2008). Quantum Dissipative Systems.

[3] Leggett AJ (1987). Dynamics of the dissipative two-state system. Rev. Mod. Phys..

[4] Zurek WH (2003). Decoherence, einselection, and the quantum origins of the classical. Rev. Mod. Phys..

[5] Zurek WH (1982). Environment-induced superselection rules. Phys. Rev. D.

[6] Lidar DA, Chuang IL, Whaley KB (1998). Decoherence-free subspaces for quantum computation. Phys. Rev. Lett..

[7] Lidar DA, Bacon D, Whaley KB (1999). Concatenating decoherence-free subspaces with quantum error correcting codes. Phys. Rev. Lett..

[8] Viola L, Lloyd S (1998). Dynamical suppression of decoherence in two-state quantum systems. Phys. Rev. A.

[9] Kofman AG, Kurizki G (2004). Unified theory of dynamically suppressed qubit decoherence in thermal baths. Phys. Rev. Lett..

[10] Jing J (2015). Nonperturbative leakage elimination operators and control of a three-level system. Phys. Rev. Lett..

[11] Zhu B (2014). Suppressing the loss of ultracold molecules via the continuous quantum zeno effect. Phys. Rev. Lett..

[12] Paavola J, Maniscalco S (2010). Decoherence control in different environments. Phys. Rev. A.

[13] Wu W, Lin H-Q (2017). Quantum zeno and anti-zeno effects in quantum dissipative systems. Phys. Rev. A.

[14] John S, Wang J (1990). Quantum electrodynamics near a photonic band gap: Photon bound states and dressed atoms. Phys. Rev. Lett..

[15] Tong Q-J, An J-H, Luo H-G, Oh CH (2010). Mechanism of entanglement preservation. Phys. Rev. A.

[16] Qiao L, Sun C-P (2019). Atom-photon bound states and non-markovian cooperative dynamics in coupled-resonator waveguides. Phys. Rev. A.

[17] Yang C-J, An J-H, Lin H-Q (2019). Signatures of quantized coupling between quantum emitters and localized surface plasmons. Phys. Rev. Res..

[18] LaHaye MD, Suh J, Echternach PM, Schwab KC, Roukes ML (2009). Nanomechanical measurements of a superconducting qubit. Nature.

[19] Hohenester U (2010). Cavity quantum electrodynamics with semiconductor quantum dots: Role of phonon-assisted cavity feeding. Phys. Rev. B.

[20] Marthaler M, Leppäkangas J (2016). Diagrammatic description of a system coupled strongly to a bosonic bath. Phys. Rev. B.

[21] Lü Z, Zheng H (2012). Communication: Engineered tunable decay rate and controllable dissipative dynamics. J. Chem. Phys..

[22] Silbey R, Harris RA (1984). Variational calculation of the dynamics of a two level system interacting with a bath. J. Chem. Phys..

[23] Irish EK, Gea-Banacloche J, Martin I, Schwab KC (2005). Dynamics of a two-level system strongly coupled to a high-frequency quantum oscillator. Phys. Rev. B.

[24] Scully MO, Zubairy MS (1997). Quantum Optics.

[25] Mahan GD (1990). Many-Partical physics.

[26] Bellomo B, Lo Franco R, Compagno G (2007). Non-markovian effects on the dynamics of entanglement. Phys. Rev. Lett..

[27] Zakian V (1969). Numerical inversion of Laplace transform. Electron. Lett..

[28] Gradshteyn IS, Ryzhik IM (2007). Table of Integrals, Series, and Products.

[29] Yang W, Ma W-L, Liu R-B (2016). Quantum many-body theory for electron spin decoherence in nanoscale nuclear spin baths. Rep. Prog. Phys..

[30] Cao X, Zheng H (2008). Non-markovian disentanglement dynamics of a two-qubit system. Phys. Rev. A.

[31] Cao X, You JQ, Zheng H, Nori F (2011). A qubit strongly coupled to a resonant cavity: Asymmetry of the spontaneous emission spectrum beyond the rotating wave approximation. New J. Phys..

[32] Gan C, Zheng H (2009). Non-markovian dynamics of a dissipative two-level system: Nonzero bias and sub-ohmic bath. Phys. Rev. E.

[33] Lü Z, Zheng H (2012). Effects of counter-rotating interaction on driven tunneling dynamics: Coherent destruction of tunneling and bloch-siegert shift. Phys. Rev. A.

[34] Wu Y (2018). An efficient and compact switch for quantum circuits. NPJ Quantum Inf..

[35] Jing J, Yu T, Lam C-H, You JQ, Wu L-A (2018). Control relaxation via dephasing: A quantum-state-diffusion study. Phys. Rev. A.

[36] Yan L-L, Zhang J-Q, Jing J, Feng M (2015). Suppression of dissipation in a laser-driven qubit by white noise. Phys. Lett. A.

[37] Fruchtman A, Lovett BW, Benjamin SC, Gauger EM (2015). Quantum dynamics in a tiered non-markovian environment. New J. Phys..

[38] Wu W, Cheng J-Q (2018). Coherent dynamics of a qubit-oscillator system in a noisy environment. Quantum Inf. Process..

[39] Tanimura Y, Kubo R (1989). Time evolution of a quantum system in contact with a nearly gaussian-markoffian noise bath. J. Phys. Soc. Jpn..

[40] an Yan Y, Yang F, Liu Y, Shao J (2004). Hierarchical approach based on stochastic decoupling to dissipative systems. Chem. Phys. Lett..

[41] Xu R-X, Yan Y (2007). Dynamics of quantum dissipation systems interacting with bosonic canonical bath: Hierarchical equations of motion approach. Phys. Rev. E.

[42] Jin J, Zheng X, Yan Y (2008). Exact dynamics of dissipative electronic systems and quantum transport: Hierarchical equations of motion approach. J. Chem. Phys..

[43] Wu W (2018). Realization of hierarchical equations of motion from stochastic perspectives. Phys. Rev. A.

[44] Wu W (2018). Stochastic decoupling approach to the spin-boson dynamics: Perturbative and nonperturbative treatments. Phys. Rev. A.

[45] Yoshihara F (2017). Superconducting qubit-oscillator circuit beyond the ultrastrong-coupling regime. Nat. Phys..

[46] Kleinekathöfer U (2004). Non-markovian theories based on a decomposition of the spectral density. J. Chem. Phys..

[47] Tang Z, Ouyang X, Gong Z, Wang H, Wu J (2015). Extended hierarchy equation of motion for the spin-boson model. J. Chem. Phys..

[48] Ritschel G, Eisfeld A (2014). Analytic representations of bath correlation functions for ohmic and superohmic spectral densities using simple poles. J. Chem. Phys..

